# The Role of FGFR1 Gene Amplification as a Poor Prognostic Factor in Squamous Cell Lung Cancer: A Meta-Analysis of Published Data

**DOI:** 10.1155/2015/763080

**Published:** 2015-12-16

**Authors:** Yang Wang, Wen Gao, Jiali Xu, Xiaojun Chen, Yang Yang, Yizhi Zhu, Yongmei Yin, Renhua Guo, Ping Liu, Yongqian Shu, Lingxiang Liu

**Affiliations:** ^1^Division of Oncology, Nanjing Medical University, Nanjing 210000, China; ^2^Department of Oncology, The First Affiliated Hospital of Nanjing Medical University, Nanjing 210000, China; ^3^College of Sciences, Nanjing University of Technology, Nanjing 210000, China; ^4^School of Public Health, Division of Biostatistics, The University of Texas Health Science Center at Houston, Houston, TX 77030, USA

## Abstract

*Objectives*. The prognostic factors of the fibroblast growth factor receptor 1 (FGFR1) in non-small cell lung cancer (NSCLC) remain controversial. *Methods*. We conducted a meta-analysis of published studies from 1974 to February 2015. In absence of quality difference between studies of reporting significant and nonsignificant results, the relationship between FGFR1 amplification and clinicopathological parameters in NSCLC was analyzed. And also the combined hazard ratio (HR) and their corresponding 95% confidence interval (CI) were calculated in terms of overall survival. *Results*. 3178 patients (12 studies) were included in the analysis. It was shown that FGFR1 amplification was significantly more prevalent among male patients (RR 2.03, 95% CI 1.57–2.63) with squamous cell lung cancer (SQCC) (RR 3.49, 95% CI 2.62–4.64) and current smokers (RR 2.63, 95% CI 1.92–3.60). The pooled data also showed that the FGFR1 amplification was a poor prognostic factor in SQCC (HR 1.38, 95% CI 1.07–1.78), Asian patients (HR 1.78, 95% CI 1.22–2.60), and fluorescence in situ hybridization (FISH) method (HR 1.30, 95% CI 1.06–1.58). *Conclusions*. This meta-analysis strongly suggests that FGFR1 amplification occurs more frequently in male, SQCC and smokers, and it is a risk factor for poor prognosis among Asian patients with SQCC.

## 1. Introduction

Lung cancer is the most common cancer in the world and also the leading cause of cancer-related mortality worldwide despite improved diagnosis and therapy. Non-small cell lung cancer (NSCLC) accounts for 84% of all lung cancer in the United States. And the 5-year overall survival rate was about 18% in NSCLC, because most of lung cancer patients were diagnosed at advanced stages [1]. Chemotherapy, which is typically the main treatment for advanced NSCLC, has achieved significant improvements in median survival time over the past decades. However, in the last decades, tobacco smoking has become a major public health challenge, especially in China [2]. Most importantly, the genetic polymorphisms were considered as the host factor contributing to tumorigenesis of NSCLC [3]. Now, oncogenic protein kinases inhibitors have been prevailing in lung adenocarcinoma, such as targeting epidermal growth factor receptor (EGFR) mutation or anaplastic lymphoma kinase (ALK) rearrangement. Unfortunately, investigation of squamous cell carcinoma (SQCC) has lagged behind, notwithstanding that the complexity of gene aberrations driving SQCC was revealed recently [4]. Among them, the fibroblast growth factor receptor 1 (FGFR1) gene, which is one of the most frequently amplified genes in human cancer, could be the most promising targets for SQCC therapy [5]. FGFR1 belongs to EGFR tyrosine kinase superfamily (FGFR1-4) [6, 7]. The ternary FGF-FGFR-HSPG (heparin sulfate proteoglycan) complex promoted transphosphorylation and then induced the further activation of several intracellular signaling cascades (RAS, P13K, etc.). So far, the FGFRs played multiple roles during cell proliferation, differentiation, antiapoptosis, stemness, embryonic development, and angiogenesis [8, 9]. Most importantly, genetic alternation of the FGFR1 led to epithelial malignancies in oral squamous, esophageal squamous, bladder, ovarian, and lung cancer [10–12]. Many researchers studied the prognostic value of FGFR1 amplification in patients with NSCLC; however inconsistency across studies and meta-analysis was found [13–15]. In order to make an updated comprehensive quantitative evaluation of the prognostic potential of FGFR1 amplification in NSCLC, we conducted a meta-analysis of updated-published literatures.

## 2. Materials and Methods

### 2.1. Search Strategy and Selection Criteria

We searched Medline, Embase, and Web of Science for articles published in English from January 1, 1974, to February 28, 2015, with the terms or combined “FGFR1”, “fibroblast growth factor receptor 1”, “lung cancer”, “lung carcinoma”, and “prognosis” and “survival”, and the references cited in the identified studies or reviews were also used to complete the search.

The inclusion criteria were the following: (1) The amplification of FGFR1 was measured in NSCLC. (2) Comparison of overall survival was done between FGFR1 amplification and nonamplified groups. (3) Hazard ratio (HR) and 95% confidence interval (CI) for overall survival according to FGFR1 status could be either reported or computed from the data or figure presented. (4) When the same author or group reported results obtained from the same patient population in more than one article, the most recent report or the most informative one was included. (5) The articles should be published as full-text papers in English. (6) Test methods included reverse transcription polymerase chain reaction (RT-PCR), fluorescent in situ hybridization (FISH), and chromogenic in situ hybridization (CISH). Two authors (Yang Wang and Yizhi Zhu) determined study eligibility independently, and disagreements were resolved by consensus.

### 2.2. Data Extraction

The clinical characteristic of patients was extracted from each study: surname of the first author, year of publication, patient ethnicity, gender, smoking status, histology, disease stage, and overall survival; FGFR1 gene copy number, test method, cutoff value, and number of cases and controls. When referring to smoking status, we defined* never smokers* as adults who never smoked or smoked fewer than 100 cigarettes in their lifetime;* former smokers* were those who smoked at least 100 cigarettes but currently do not smoke;* current smokers* were people who smoked 100 cigarettes in their lifetime and currently smoke.* Nonsmokers* were defined as both former smokers and never smokers [16]. HR as well as 95% CI for survival was either directly from the manuscript if available or calculated from survival curve [17]. Two authors read the curves independently to minimize inaccuracy.

### 2.3. Methodological Assessment

Two authors independently read and scored each study according to the ELCWP (European Lung Cancer Working Party) scale established by Steels et al. [18]. The global quality score was assessed according to 4 main categories: (1) the scientific design; (2) the description of the methods used to identify the amplification of FGFR1; (3) the generalizability of the results; (4) the data analysis. Each category had a maximal score of 10 points; therefore the overall maximum score was 40 points. Finally the scores were expressed as percentages ranging from 0% to 100%. The higher the score was, the better the methodological quality indicated.

### 2.4. Definition of Outcomes and Comparisons

The comprehensive analysis of the relationship was conducted between FGFR1 amplification and histology, gender, smoking status, and stage, using relative risk (RR). Furthermore, the overall HR (95% CI) was estimated by individual HR (95% CI), and HR (95% CI) >1 implied a poor prognosis associated with FGFR1 amplification. In addition, the subgroups were evaluated including histology, ethnics, and test method.

### 2.5. Statistical Analysis

Statistical heterogeneity was evaluated with the Chi-square based *Q*-test [19] and *I* ^2^ statistic, and significant heterogeneity was determined when *p* < 0.1. The modest to low heterogeneity across studies was identified if *I* ^2^ ≤ 50%, and then the fixed-effects model was used. If *I* ^2^ ≥ 50% with the high heterogeneity, we would calculate based on the random-effects model [20]. The significance of the pooled HR was determined by the *Z*-test and *p* < 0.05 was considered statistical significance. Publication bias was assessed by Egger's regression and Begg's funnel plot [21], while *p* ≤ 0.1 was set as statistical significance. Statistical computations were all performed with Stata v10.0 (Stata Corporation, TX, USA). All *p* values were two-sided.

## 3. Results

### 3.1. Trial Flow

**Figure 1:**
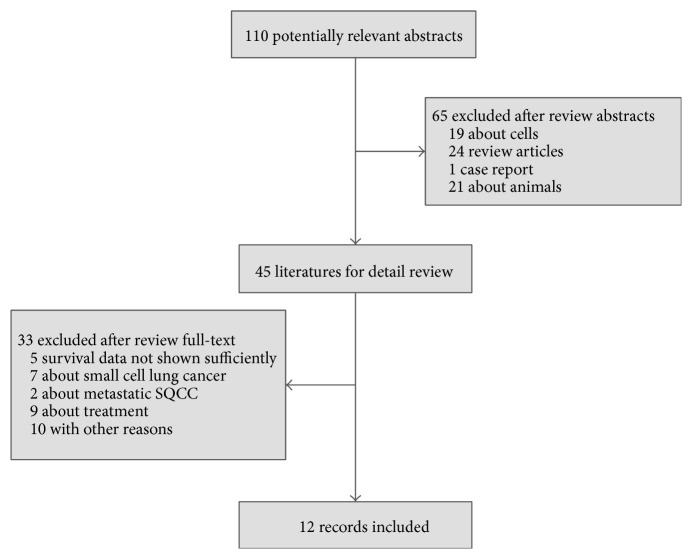
Flow diagram of search results.

Figure 1 and Table 1 depicted results of the literature search, containing major characteristics of the study population. From January 1, 1974, to February 28, 2015, 3178 patients (12 studies) were identified and included in the final analysis. 5 studies were excluded due to the incomplete prognostic values of FGFR1 amplification. 9 articles were excluded due to alternative diagnoses (7 for small cell lung cancer and 2 focusing on metastatic SQCC).

### 3.2. Study Characteristics

The median sample size across 12 studies is 263 (100–445) in this analysis. 3 studies (23.0%, 731/3178) were conducted in Asian and 9 studies (77.0%, 2447/3178) in non-Asian. Gene copy number of FGFR1 was evaluated by FISH in 9 studies (77.7%, 2469/3178), by PCR in 2 studies (14.0%, 445/3178) [22, 23], and by CISH in 1 study (8.3%, 264/3178) [24]. Males account for 71.2% of the patients (2002/2813), SQCC for 57.4% (1823/3178), and current smokers for 56.2% (1141/2030). 11.8% (375/3178) patients were identified with FGFR1 amplification. 3 studies (29.5%, 936/3178) indicated that FGFR1 amplification was an independent factor for poor prognosis [22, 25, 26], and only 1 study (8.3%, 264/3178) favored overall survival [24]. Moreover no significant prognostic impact of FGFR1 was found in 8 lung cancer studies (62.2%, 1978/3178).

### 3.3. Assessment of Study Quality

The median value of global quality score was 57.5% (42.5%–82.5%). Among them, scientific design and laboratory methodology got the highest value (median, 6.4/10) and the lowest value was result analysis (median, 4.4/10). No quality difference was found between studies reporting significant and nonsignificant results (*p* = 0.083) (Table S1, in Supplementary Material available online at http://dx.doi.org/10.1155/2015/763080).

### 3.4. Test of Heterogeneity

The heterogeneity was analyzed for all the included 12 studies between FGFR1 amplification and overall survival, with the Chi-square test (*I* ^2^ = 55.4%, *p* = 0.008) in a random-effects model, indicating there was some heterogeneity between studies. More importantly, in the subgroup analysis of SQCC, Asian, and test method, FISH, no significant heterogeneity was detected (Table S2).

### 3.5. Meta-Analysis

**Figure 2:**
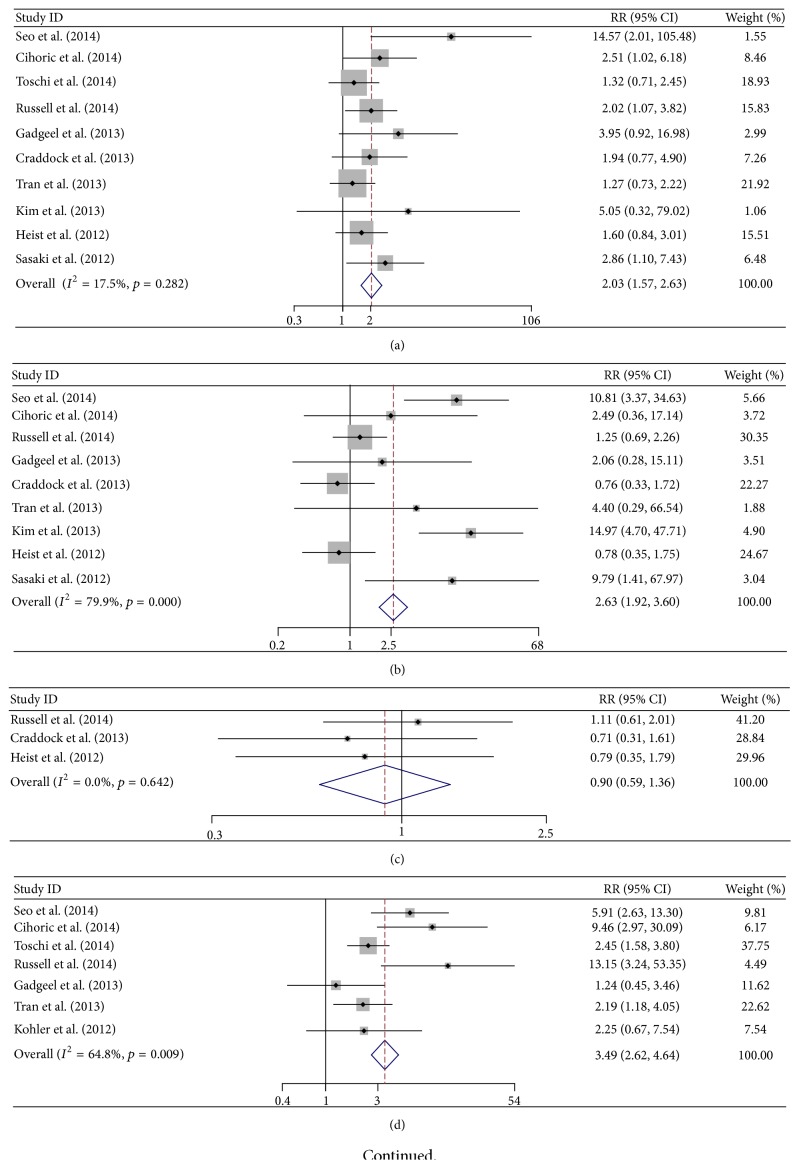
Meta-analysis of relative risk (RR) of FGFR1 in diffenent characteristics. (a) FGFR1 amplification in different gender (male versus female); (b) FGFR1 amplification in current smokers versus nonsmokers; (c) FGFR1 amplification in current smokers versus former smokers; (d) FGFR1 amplification in different histology (SQCC versus ADE); (e) FGFR1 amplification in different stages (stages III-IV versus I-II).

**Figure 3:**
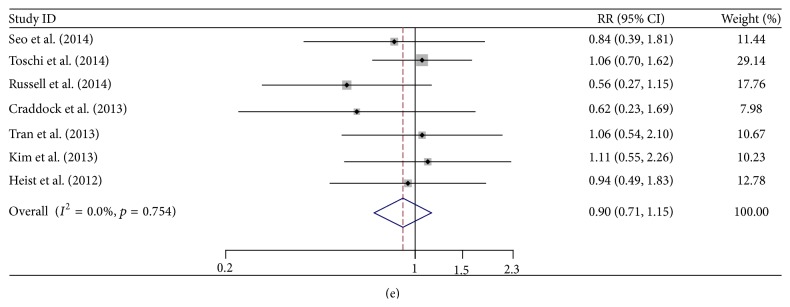
Meta-analysis of the hazard ratio (HR) about the relationship of the FGFR1 amplification and overall survival. (a) All the 12 included studies (NSCLC); (b) subgroup of different histology (NSCLC after removing all of the known SQCC; SQCC); (c) subgroup of different ethnics (Asian; non-Asian); (d) studies using test method, FISH.

As for the relationship between FGFR1 amplification and clinical characteristics (histology, gender, smoking status, and stage), it showed that FGFR1 amplification occurred more frequently in males (RR 2.03, 95% CI 1.57–2.63), SQCC (RR 3.49, 95% CI 2.62–4.64), and current smokers (RR 2.63, 95% CI 1.92–3.60). However, no significant difference was found between different stages in NSCLC (RR 0.90, 95% CI 0.71–1.15) (Tables 2 and 3, Figure 2). The results of each meta-analysis were presented in Figure 3. The combined HR of 12 studies (3178 patients), evaluating the association of FGFR1 amplification and overall survival, was 1.30 (95% CI, 1.01–1.67). However no significance was found after removing all of the SQCC (HR 1.32, 95% CI 0.91–1.93). More importantly, in subgroup analyses, it was indicated that FGFR1 amplification was a significant poor prognostic factor in SQCC (all by FISH method, HR 1.38, 95% CI 1.07–1.78) and Asian (HR 1.78, 95% CI 1.22–2.60). In order to minimize bias of test methods, 3 studies with non-FISH methods were excluded for the sensitivity analysis. Interestingly, FGFR1 amplification also showed poor prognostic significance (HR 1.30, 95% CI 1.06–1.58).

### 3.6. Publication Bias

Begg's funnel plot and Egger's regression test were applied for detecting publication bias in the meta-analysis. In all included studies, no funnel plot asymmetry was found (*p* = 0.152), and 95% CI was −0.79–4.43 in Egger's test. Therefore, there is no evident publication bias in the analysis (Figure S1).

## 4. Discussion

It was reported that the FGFR1 amplification could be detected in about 10%–20% of the lung SQCC, with lower frequency in 1%–5% of lung adenocarcinoma [25, 27, 28], which was consistent with the results of our meta-analysis.

As for the correlation of FGFR1 amplification with clinical features, it was found that the frequency of FGFR1 amplification increased in smokers [23]. And what is more in a smoking dosage dependent manner is that the frequency of FGFR1 amplification significantly increased with smoking pack-year history, and the smoking index of FGFR1 amplification (40 pack-years) was significantly higher than that of FGFR1 disomy or the low-amplification group (30 pack-years; *p* = 0.01) [25, 29]. Furthermore, our systematic analysis also showed that FGFR1 amplification was more frequent in current smokers (median 17.4%, 5.6%–40.8%) than nonsmokers (median 4.2%, 0.0%–21.5%), but no statistical difference was found in the subgroup analysis of current/former smokers. Additionally, the frequency of FGFR1 amplification was significantly higher in males (median 17.2%, 6.6%–39.4%) than females (median 10.0%, 0.0%–15.8%) and much higher in SQCC (median 18.0%, 5.1%–28.3%) than lung adenocarcinoma (median 4.1%, 1.7%–11.5%).

Despite large genomic study, “driver” gene should be linked to lung cancer outcomes, pooling scientific data reported from multiple studies. Some meta-analysis concerning FGFR1 had been reported; however no exact results can be provided due to large sample size studies (*n* = 1973 [22, 26, 28–31]) published recently [15], confusing SQCC with NSCLC in HR [14] and focusing on various squamous cell cancers instead of lung cancer alone [13]. A set of criteria and collecting comprehensive data from all articles published in English until late February 2015 make our data more reliable. In order to find out the source of heterogeneity, we make subgroup analysis including histology (SQCC, *I* ^2^ = 2.0%, *p* = 0.395), ethnics (Asian, *I* ^2^ = 0.0%, *p* = 0.937), and test method (FISH, *I* ^2^ = 16.3%, *p* = 0.298) (Table S1). It indicated that the same histological patterns, test methods, and ethnics could eliminate the heterogeneity. Most importantly, in the subgroup of SQCC (*n* = 897), with the consistency of test method, FISH, significant prognostic value (HR 1.38, 1.07–1.78) was found without heterogeneity. And also it was found in the subgroup of test method, FISH (*n* = 2469, HR 1.30, 1.06–1.58), and Asian (*n* = 731, HR 1.78, 1.22–2.60) (Figure 3). Moreover, no significant correlation was found between FGFR1 amplification and prognosis of NSCLC in the analysis after removing all of the SQCC. The FGFR1 drives stronger downstream pathway activation than other FGFRs [32], and its amplification has been verified as independent prognostic factor in some cancers including breast cancer [33, 34]. So, it strongly suggested that FGFR1 was a potential target for SQCC, and it will add a new member besides PIK3CA [4] and DDR2 [35].

Otherwise, FGFR1 protein expression, uncorrelated with patient outcome, did not show any relationship with FGFR1 amplification [29, 36, 37]. In contrast, Kim et al. demonstrated a strong correlation between FGFR1 amplification and mRNA/protein expression [25]. Systematic analysis cannot be done in this meta-analysis because there is not enough data about FGFR1 expression and prognosis. Moreover, FGFR1 amplification of primaries was highly concordant with lymph node metastases (97.7%) [38], so doing a biopsy on metastatic cancer may make sense in order to determine the FGFR1 status of the primary tumor. With regard to the predictive value for treatment in lung cancer, it also found that patients with FGFR1 amplification could benefit from adjuvant chemotherapy [25], but it cannot be validated in our analysis for lacking of more studies in lung cancer.

Certainly, caution should be taken into account about biases. First, publication bias is a major concern in the meta-analysis. Although the summary statistics did not support publication bias, only English articles included could not completely avoid language bias. Second, the cutoff values of gene copy number in each study varied from 2 to 9 (median = 4); it should be well-controlled in the future research in order to avoid the same mistakes as ERCC1 [39]. Third, the correlation of the smoking index and FGFR1 amplification remained elusive, because only four articles included in our analysis referred to pack-year index [22, 25, 29, 40] (Table S3). Last but not least, we also compare the clinical features between studies reporting significant and nonsignificant results, and no significant differences were found (data not shown). Methodological assessment was conducted to avoid selection bias, and no statistical difference was found between positive and negative studies. Then, it boosts our confidence in the analysis.

In conclusion, this is the updated comprehensive analysis to show that the FGFR1 amplification can be a poor prognostic marker in SQCC. Although well-designed prospective validation and individual participant data (IPD) based analysis is warranted, a number of FGFR inhibitors, such as BGJ398, Brivanib, Dovitinib, Nintedanib, and Orantinib, are already underway in several solid tumors. This study provides a rationale for inclusion of SQCC patients in such studies.

## Supplementary Material

Table S1: Quality of the selected literatures was assessed using the European Lung Cancer Working Party scale. Overall, the median value of global quality score was 57.5% (42.5%-82.5%). No quality difference was found between studies reporting significant and nonsignificant results.
Table S2: The heterogeneity was analyzed for all the 12 studies between FGFR1 amplification and overall survival with the Chi-square test. There was some heterogeneity when analyzing all the studies and in the subgroup of NSCLC. However, in the subgroup analysis of SQCC, Asian, and test method-FISH, no significant heterogeneity was detected.
Table S3: 4 papers refers to the pack-year (PY) of smoking. In Kim et al.'s paper , the smoking dosage of FGFR1 amplification (40 PY) was significantly higher than that of FGFR1 disomy group or the low-amplification group (30 PY; p=0.01). In Seo et al.'s paper, the frequency of FGFR1 amplification significantly increased with cigarette smoking pack-year history in NSCLC (p<0.001) and in adenocarcinoma (p=0.021). In Gadgeel et al.'s study, no statistically significant difference was found for FGFR1 amplification between tumors of patients with “light” (<15PY) and “heavy” (≥15PY) (p=0.74). And also in Heist et al.'s paper, no significant correlation was found between FGFR1 amplification and PY history in SqCC (p=0.79).
Figure S1: The Begg's funnel plot and Egger's regression test was applied for detecting publication bias. no funnel plot asymmetry was found (p=0.152), and 95%CI was -0.79-4.43 in Egger's test, Indicating that there is no evident publication bias in the analysis.

## Figures and Tables

**Table 1:** Characteristics of the studies included in the meta-analysis.

Author	Year	Race	NP	Positive (number)	Positive (%)	Histology	Stage	Test method	HR estimate	Cutoff	Result
Seo et al. [29]	2014	Japan	369	33	8.9	NSCLC	I–III	FISH	HR + CI	≥6.2	NS
Cihoric et al. [26]	2014	Switzerland	329	41	12.5	NSCLC	I-II	FISH	HR + CI	≥2	Poor
Toschi et al. [30]	2014	America	445	74	16.6	NSCLC	I–III	FISH	Survival curves	≥4	NS
Russell et al. [31]	2014	Australia	352	50	14.2	NSCLC	I–IV	FISH	HR + CI	≥2.0	NS
Gadgeel et al. [22]	2013	America	345	12	3.5	NSCLC	I–IV	qPCR	HR + CI	>3.5	Poor
Craddock et al. [38]	2013	Canada	121	11	9.1	SQCC	I–IV	FISH	HR + CI	≥5	NS
Tran et al. [24]	2013	Australia	264	37	14.0	NSCLC	I–IV	CISH	Survival curves	≥2.0	Favor
Kim et al. [25]	2013	Korean	262	34	13.0	SQCC	I–III	FISH	HR + CI	≥9	Poor
Heist et al. [40]	2012	America	226	37	16.4	SQCC	I–IV	FISH	Survival curves	≥2.2	NS
Kohler et al. [28]	2012	Germany	133	14	10.5	SQCC	—	FISH	Survival curves	≥4	NS
Sasaki et al. [23]	2012	Japan	100	32	32.0	NSCLC	I–IV	qPCR	Survival curves	>4	NS
Weiss et al. [27]	2010	Germany	232	16	6.9	NSCLC	I–III	FISH	Survival curves	>9	NS

NP, number of patients; HR, hazard ratio; CI, confidence interval; NSCLC, non-small cell lung cancer; SQCC, squamous cell lung cancer; FISH, fluorescence in situ hybridization; qPCR, quantitative polymerase chain reaction; CISH, chromogenic in situ hybridization; NS, nonsignificant.

**Table 2:** Patients' number of clinical characteristics.

	Histology		Gender		Smoking			pStage	
	SQCC	ADE	Male	Female	C	N	F	III-IV	I-II
Seo et al. [29]	139	230	251	118	127	142	—	264	105
Cihoric et al. [26]	169	137	244	85	239	18	—	—	—
Toschi et al. [30]	138	243	369	76	—	—	—	265	180
Russell et al. [31]	178	117	222	130	69	258	223	261	91
Gadgeel et al. [22]	136	169	226	119	270	37	—	—	—
Craddock et al. [38]	—	—	77	44	43	65	61	89	32
Tran et al. [24]	101	115	169	95	165	9	—	223	41
Kim et al. [25]	—	—	245	17	107	155	—	198	64
Heist et al. [40]^*∗*^	—	—	128	98	45	181	172	155	61
Kohler et al. [28]	133	64	—	—	—	—	—	—	—
Sasaki et al. [23]	—	—	71	29	76	24	—	—	—
Weiss et al. [27]	—	—	—	—	—	—	—	—	—
Overall	—	—	—	—	—	—	—	—	—

SQCC, squamous cell lung cancer; ADE, lung adenocarcinoma; C, current smokers; N, never smokers; F, former smokers; pStage, pathological stage.

^*∗*^Pathological stage or clinical stage, not specified.

**Table 3:** RRs of FGFR1 amplification in the studies according to different histology, gender, smoking status, and stage.

	RRs (95% CI)				
	Histology (SQCC versus ADE)	Gender (M versus F)	Current smokers versus nonsmokers	Current smokers versus former smokers	pStage (III-IV versus I-II)
Seo et al. [29]	**5.91 (2.63–13.30)**	**14.57 (2.01–105.48)**	**10.81 (3.37–34.63)**	—	0.84 (0.39–1.81)
Cihoric et al. [26]	**9.46 (2.97–30.09)**	**2.51 (1.02–6.18)**	2.49 (0.36–17.14)	—	—
Toschi et al. [30]	**2.45 (1.58–3.80)**	1.32 (0.71–2.45)	—	—	1.06 (0.70–1.62)
Russell et al. [31]	**13.15 (3.24–53.35)**	**2.02 (1.07–3.82)**	1.25 (0.69–2.26)	1.11 (0.61–2.01)	0.56 (0.27–1.15)
Gadgeel et al. [22]	1.24 (0.45–3.46)	3.95 (0.92–16.98)	2.06 (0.28–15.11)	—	—
Craddock et al. [38]	—	1.94 (0.77–4.90)	0.76 (0.33–1.72)	0.71 (0.31–1.61)	0.62 (0.23–1.69)
Tran et al. [24]	**2.19 (1.18–4.05)**	1.27 (0.73–2.22)	4.40 (0.29–66.54)	—	1.06 (0.54–2.10)
Kim et al. [25]	—	5.05 (0.32–79.02)	**14.97 (4.70–47.71)**	—	1.11 (0.55–2.26)
Heist et al. [40]^*∗*^	—	1.60 (0.84–3.01)	0.78 (0.35–1.75)	0.79 (0.35–1.79)	0.94 (0.49–1.83)
Kohler et al. [28]	2.25 (0.67–7.45)	—	—	—	—
Sasaki et al. [23]	—	**2.86 (1.10–7.43)**	**9.79 (1.41–67.97)**	—	—
Weiss et al. [27]	—	—	—	—	—
Overall	**3.49 (2.62–4.64)**	**2.03 (1.57–2.63)**	**2.63 (1.92–3.60)**	0.90 (0.59–1.36)	0.90 (0.71–1.15)

RR, relative risk; SQCC, squamous cell lung cancer; ADE, lung adenocarcinoma; M, male; F, female; pStage, pathological stage.

^*∗*^Pathological stage or clinical stage, not specified.
