# Supplementary material for: The Role of FGFR1 Gene Amplification as a Poor Prognostic Factor in Squamous Cell Lung Cancer: A Meta-Analysis of Published Data
Source: Biomed Res Int. 2015 Dec 16;2015:763080. doi: 10.1155/2015/763080 (PMC4695660; doi:10.1155/2015/763080)
Supplement: Supplementary file 1 — Table S1: Quality of the selected literatures was assessed using the European Lung Cancer Working Party scale. Overall, the median value of global quality score was 57.5% (42.5%-82.5%). No quality difference was found between studies reporting significant and nonsignificant results. Table S2: The heterogeneity was analyzed for all the 12 studies between FGFR1 amplification and overall survival with the Chi-square test. There was some heterogeneity when analyzing all the studies and in the subgroup of NSCLC. However, in the subgroup analysis of SQCC, Asian, and test method-FISH, no significant heterogeneity was detected. Table S3: 4 papers refers to the pack-year (PY) of smoking. In Kim et al.'s paper , the smoking dosage of FGFR1 amplification (40 PY) was significantly higher than that of FGFR1 disomy group or the low-amplification group (30 PY; p=0.01). In Seo et al.'s paper, the frequency of FGFR1 amplification significantly increased with cigarette smoking pack-year history in NSCLC (p<0.001) and in adenocarcinoma (p=0.021). In Gadgeel et al.'s study, no statistically significant difference was found for FGFR1 amplification between tumors of patients with “light” (<15PY) and “heavy” (≥15PY) (p=0.74). And also in Heist et al.'s paper, no significant correlation was found between FGFR1 amplification and PY history in SqCC (p=0.79). Figure S1: The Begg's funnel plot and Egger's regression test was applied for detecting publication bias. no funnel plot asymmetry was found (p=0.152), and 95%CI was -0.79-4.43 in Egger's test, Indicating that there is no evident publication bias in the analysis. [file 763080.f1.doc]

TABLE S1: Results of methodological assessment by European Lung Cancer Working Party score

|  | Scientific design | Laboratory methodology | Generalizability | Results analysis | Overall score | Reporting  result* |
| --- | --- | --- | --- | --- | --- | --- |
| Seo(2014) | 6 | 6 | 5 | 5 | 55.00% | NS |
| Cihoric(2014) | 7 | 6 | 6 | 5 | 60.00% | S |
| Toschi(2014) | 6 | 5 | 5 | 4 | 50.00% | NS |
| Russell(2014) | 7 | 7 | 6 | 5 | 62.50% | NS |
| Gadgeel(2013) | 7 | 7 | 6 | 4 | 60.00% | S |
| Craddock(2013) | 7 | 6 | 6 | 5 | 60.00% | NS |
| Tran(2013) | 6 | 6 | 6 | 4 | 55.00% | S |
| Kim(2013) | 8 | 10 | 10 | 5 | 82.50% | S |
| Heist(2012) | 6 | 5 | 5 | 4 | 50.00% | NS |
| Kohler(2012) | 7 | 6 | 6 | 5 | 60.00% | NS |
| Sasaki(2012) | 6 | 8 | 4 | 3 | 52.50% | NS |
| Weiss(2010) | 4 | 5 | 4 | 4 | 42.50% | NS |
| Average | 6.4 | 6.4 | 5.8 | 4.4 | 57.50% | - |

*Levene's Test for Equality of Variances, p=0.213;

Significant (S) vs. nonsignificant (NS) result, t-test, *p*=0.083.

TABLE S2: Heterogeneity of the 12 evaluable studies assessing FGFR1 in NSCLC

|  | I^2^ | *p* |
| --- | --- | --- |
| Overall (n = 12) | 55.4% | 0.008 |
|  |  |  |
| SQCC (n = 5) | 2.0% | 0.395 |
| NSCLC (n = 7) | 66.5% | 0.004 |
|  |  |  |
| Non-Asian(n = 9) | 58.8% | 0.009 |
| Asian(n = 3) | 0.0% | 0.937 |
|  |  |  |
| Method with FISH(n = 9) | 16.3% | 0.298 |

SQCC, squamous cell lung cancer; NSCLC, non-small cell lung cancer; FISH, fluorescence in situ hybridization.

TABLE S3: Correlation of pack-year of smoking index and FGFR1 amplification in the 12 evaluable studies

| Author | PY correlated | Notes |
| --- | --- | --- |
| Seo(2014) | Yes | In NSCLC (p<0.001), in Adenocarcinoma (p=0.021) |
| Cihoric(2014) | NA | NA |
| Toschi(2014) | NA | NA |
| Russell(2014) | NA | NA |
| Gadgeel(2013) | No | Cutoff=15PY, p=0.74 |
| Craddock(2013) | NA | NA |
| Tran(2013) | NA | NA |
| Kim(2013) | Yes | In FGFR1 amplification group (median 40PY), in low amplification or disomy group (median 30PY) (p=0.01) |
| Heist(2012) | No | In FGFR1 amplification group (median 53PY), in not amplification group (median 50PY) (p=0.79) |
| Kohler(2012) | NA | NA |
| Sasaki(2012) | NA | NA |
| Weiss(2010) | NA | NA |

PY, pack-year; NSCLC, non-small cell lung cancer; NA, not available.

FIGURE S1: Contour-enhanced funnel plot of the 12 evaluable studies assessing FGFR1 in NSCLC.
